# Proteomic Analysis of Adrenocorticotropic Hormone Treatment of an Infantile Spasm Model Induced by *N*-Methyl-d-Aspartic Acid and Prenatal Stress

**DOI:** 10.1371/journal.pone.0045347

**Published:** 2012-09-18

**Authors:** Jing Wang, Juan Wang, Ying Zhang, Guang Yang, Wen-Jing Zhou, Ai-Jia Shang, Li-Ping Zou

**Affiliations:** 1 Department of Pediatrics, General Hospital of Chinese People's Liberation Army, Beijing, China; 2 Department of Neurosurgery, Tsinghua University Yuquan Hospital, Beijing, China; Beijing Institute of Radiation Medicine, China

## Abstract

Infantile spasms is an age-specific epileptic syndrome associated with poor developmental outcomes and poor response to nearly all traditional antiepileptic drugs except adrenocorticotropic hormone (ACTH). We investigated the protective mechanism of ACTH against brain damage. An infantile spasm rat model induced by *N*-methyl-d-aspartate (NMDA) in neonate rats was used. Pregnant rats were randomly divided into the stress-exposed and the non-stress exposed groups, and their offspring were randomly divided into ACTH-treated spasm model, untreated spasm model, and control groups. A proteomics-based approach was used to detect the proteome differences between ACTH-treated and untreated groups. Gel image analysis was followed by matrix-assisted laser desorption/ionization time-of-flight mass spectrometric protein identification and bioinformatics analysis. Prenatal stress exposure resulted in more severe seizures, and ACTH treatment reduced and delayed the onset of seizures. The most significantly up-regulated proteins included isoform 1 of tubulin β-5 chain, cofilin-1 (CFL1), synaptosomal-associated protein 25, malate dehydrogenase, N(G),N(G)-dimethylarginine dimethylaminohydrolase 1, annexin A3 (ANXA3), and rho GDP-dissociation inhibitor 1 (ARHGDIA). In contrast, tubulin α-1A chain was down-regulated. Three of the identified proteins, ARHGDIA, ANXA3, and CFL1, were validated using western blot analysis. ARHGDIA expression was assayed in the brain samples of five infantile spasm patients. These proteins are involved in the cytoskeleton, synapses, energy metabolism, vascular regulation, signal transduction, and acetylation. The mechanism underlying the effects of ACTH involves the molecular events affected by these proteins, and protein acetylation is the mechanism of action of the drug treatment.

## Introduction

Infantile spasms (West syndrome) is an age-specific epileptic syndrome associated with many underlying conditions. It is often related to poor developmental outcomes, including severe cognitive dysfunction, brain damage, and mental regression [1]. This disease responds poorly to nearly all traditional antiepileptic drugs, except adrenocorticotropic hormone (ACTH); however, the mechanisms of action of ACTH are still unclear. The conventional hypothesis states that ACTH modulates the expression and release of several neurotransmitters and neuromodulators by promoting glucocorticoid release, as well as by directly modulating amygdala neurons to decrease the proconvulsant peptide CRH [2]. The protective mechanism of ACTH against brain damage was investigated using two-dimensional electrophoresis (2DE) to analyze the proteomic changes following ACTH treatment in an animal model.

The *N*-methyl-d-aspartic acid (NMDA) model stimulates attacks in developing and young rats, causing rat hyperflexion seizures such as infantile spasms. However, ACTH has low efficiency in seizure models that involve non-stressed brains [2]–[4]. Using a case-control study, we found that the degree of prenatal stress (PS) is higher among mothers of infantile spasm patients than among those of the control group and that within a specific range, the risk of infantile spasms increases with the degree of PS [5]. Animal studies have shown that PS significant affects the subsequent physical and mental health of the offspring and it plays an important role in increasing seizure vulnerability [6]. Therefore, we hypothesized that prenatal stress exposure improves the effectiveness of ACTH in our model. Pregnant rats were forced to swim in cold water to induce PS [7]. The severity of their spasms was assessed, and the effectiveness of ACTH therapy was evaluated.

## Results

### Animal Model

All NMDA-treated rats exhibited a well-defined behavioral pattern characterized by shortness of breath, irregular and dull eyes, a slight facial muscle twitch, shaking of the jaw, shaking of the head, frequent flicking of the tail, twisting of the body, moving around/irritability, biting other animals, and a high degree of paroxysmal spasms. Rat behavior was observed over time. The severity of the spasms in the animal models was assessed using spasm frequency and spasm latency (Table 1).

**Table 1:** Degree of spasm in the animal models.

Group	Spasm frequency (n)	Spasm latency (min)
A1 PS-spasm model-ACTH treatment	23±5	29±6
A2 PS-spasm model*	33±5	17±3
B1 spasm model-ACTH treatment	24±5	30±4
B2 spasm model	22±2	21±3
B3 control	–	–

* 14 rats died from spasm.

**Figure 1:**
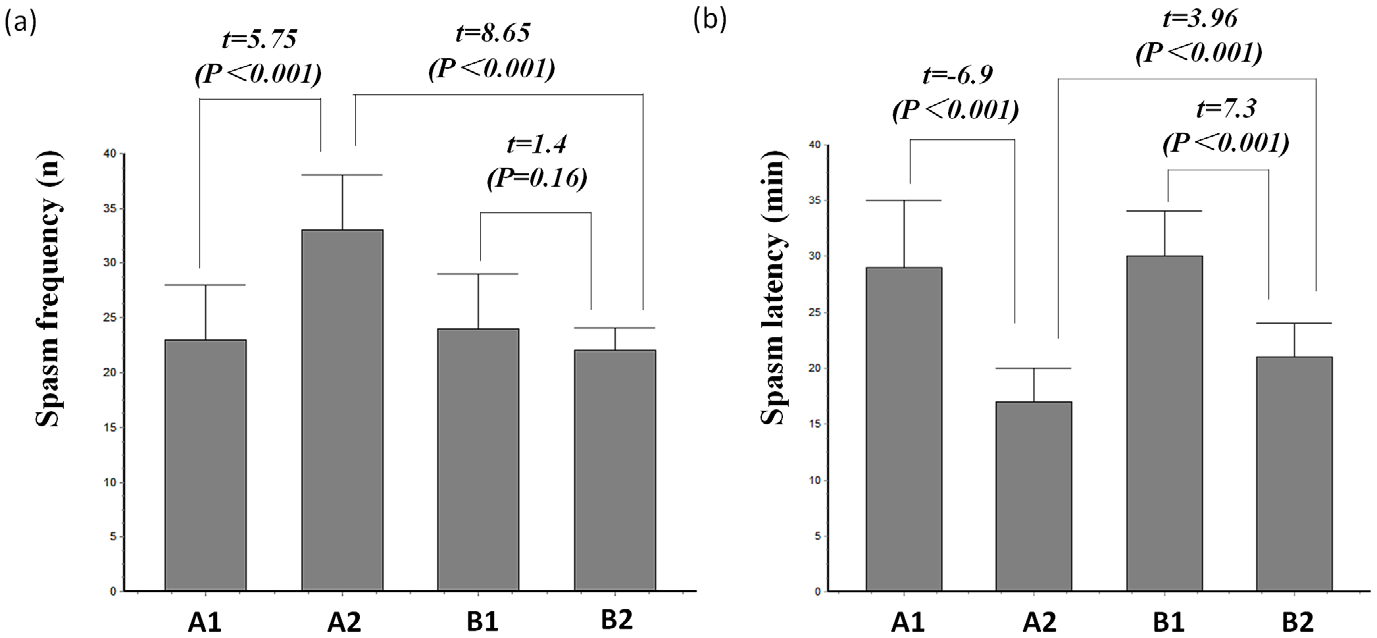
Severity of spasm in the animal models. (a) spasm frequency, (b) spasm latency. Group A1, prenatal stress (PS)-spasm model-ACTH treatment; Group A2, PS-spasm model; Group B1, spasm model-ACTH treatment; and Group B2, spasm model. The PS-spasm model (Group A2 vs. Group B2) showed an increased spasm frequency (t = 8.65, *P<*0.001) and a shortened latency period (t = 3.96, *P*<0.001). ACTH treatment was effective in treating the PS-spasm model (Group A1 vs. Group A2) and shows a lower spasm frequency (t = 5.75, *P*<0.001) and a lengthened latency period (t = 6.9, *P*<0.001). The spasm models showed no obvious response to ACTH treatment (Group B1 vs. Group B2), but a lengthened latency period was observed (t = 7.3, *P*<0.001). Spasm frequency was not significantly different among the models (t = 1.4, *P* = 0.16).

Compared with the spasm model (Figure 1), the PS-spasm model exhibited an increased spasm frequency (t = 8.65, *P*<0.001) and a shorter latency period (t = 3.96, *P*<0.001). ACTH treatment was effective in the PS-spasm model, which exhibited a lower spasm frequency (t = 5.75, *P*<0.001) and longer latency period (t = 6.9, *P*<0.001). However, the spasm models did not show obvious responses to ACTH treatment; the latency period was longer (t = 7.3, *P*<0.001), but no improvement was observed in spasm frequency (t = 1.4, *P* = 0.16). Based on these results, we concluded that maternal stress during pregnancy results in a higher number of severe seizures in the animal models. ACTH treatment reduced onset, which suggests a response to ACTH treatment.

### 2DE Analysis

Based on the 2DE maps that contained 46 spots, a ±1.5-fold difference was observed in the prenatal stress-spasm model-ACTH treatment (Group A1) compared with the PS-spasm model (Group A2). A total of 10 protein spots represented up-regulated proteins, whereas 36 represented down-regulated proteins.

**Figure 2:**
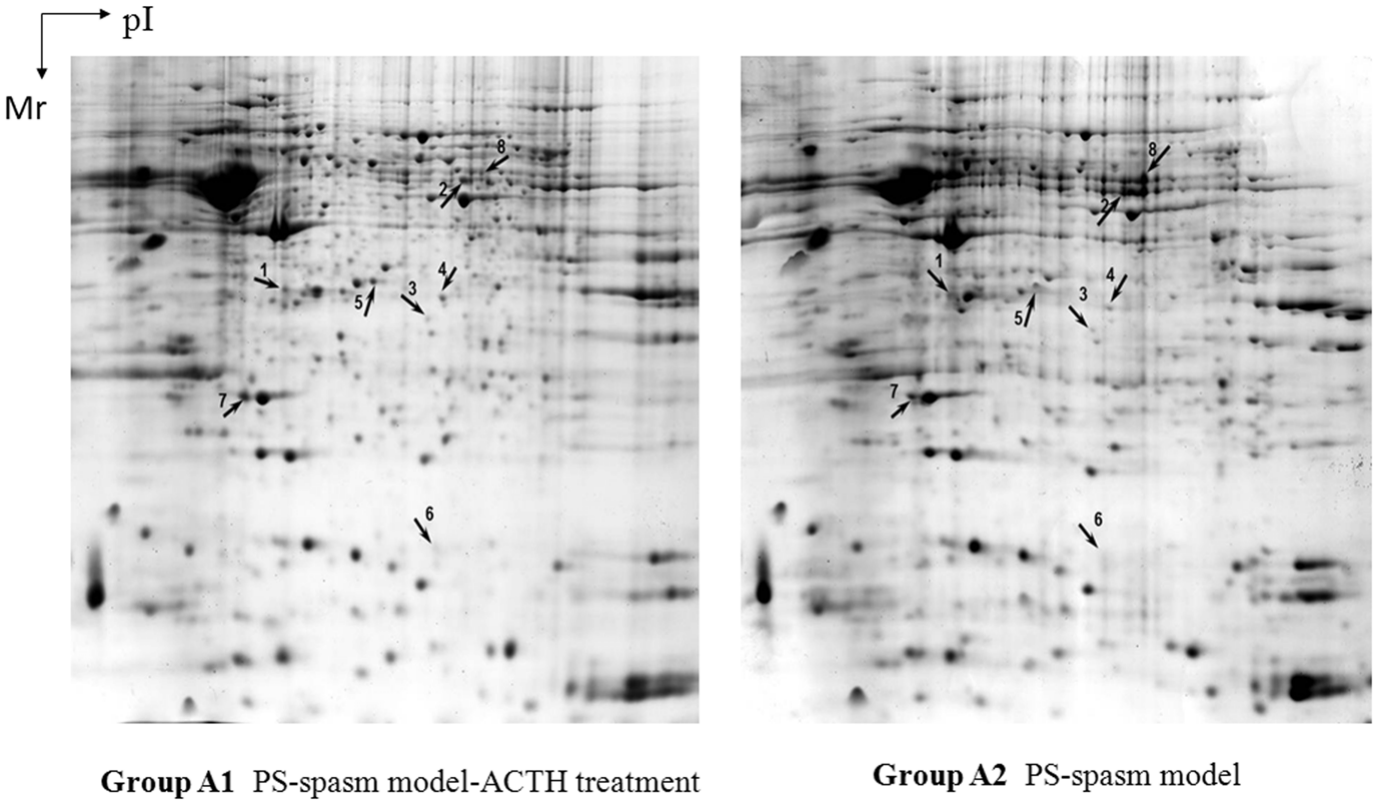
2DE map of the rat brain from Groups A1 and A2 stained with CBB. The gels were loaded with 1 mg of protein, run on 24 cm pH 3–10 NL IPG, and separated using 12% SDS-PAGE. Numbered protein spots were identified.

Eight differentially expressed proteins were identified. These proteins are shown in Figure 2 and their identification results are in Table 2. The differentially expressed proteins are involved in various biological functions, including cytoskeletal rearrangement (tubulin α-1A chain, isoform 1 of tubulin β-5 chain, n-cofilin [CFL1]), neuroprotection (*N*(G),*N*(G)-dimethylarginine dimethylaminohydrolase [Ddah1], synaptosomal-associated protein 25 [SNAP-25]), and metabolism (malate dehydrogenase [Mdh1], rho GDP-dissociation inhibitor 1 [ARHGDIA]).

**Table 2:** Significantly differentially expressed proteins identified from rat brain samples using MALDI-TOF/TOF-MS.

Rank	Symbol/Description	Accession No.	Mr	pI	Score	Pep. Count	Fold Change
**Up-regulated proteins**							
1	Snap25/Synaptosomal-associated protein 25	IPI00204644	129676	9.39	71	18	2.81
2	Tubb5/Isoform 1 of the Tubulin β-5 chain	IPI00197579	49639	4.78	97	10	2.74
3	Anxa3/Annexin A3	IPI00207390	36341	5.96	225	16	2.56
4	Mdh1/Malate dehydrogenase, cytoplasmic	IPI00198717	36460	6.16	151	12	1.76
5	Ddah1/N(G),N(G)-dimethylarginine dimethylaminohydrolase 1	IPI00231194	31406	5.75	153	17	1.75
6	Cfl1/Cofilin-1	IPI00327144	18521	8.22	77	14	1.56
7	Arhgdia/Rho GDP-dissociation inhibitor 1	IPI00196994	23393	5.12	182	11	1.53
**Down-regulated proteins**							
8	Tuba1a/Tubulin α-1A chain	IPI00189795	50104	4.94	98	17	0.48

### Validation of Differential Expression by Western Blot

**Figure 3:**
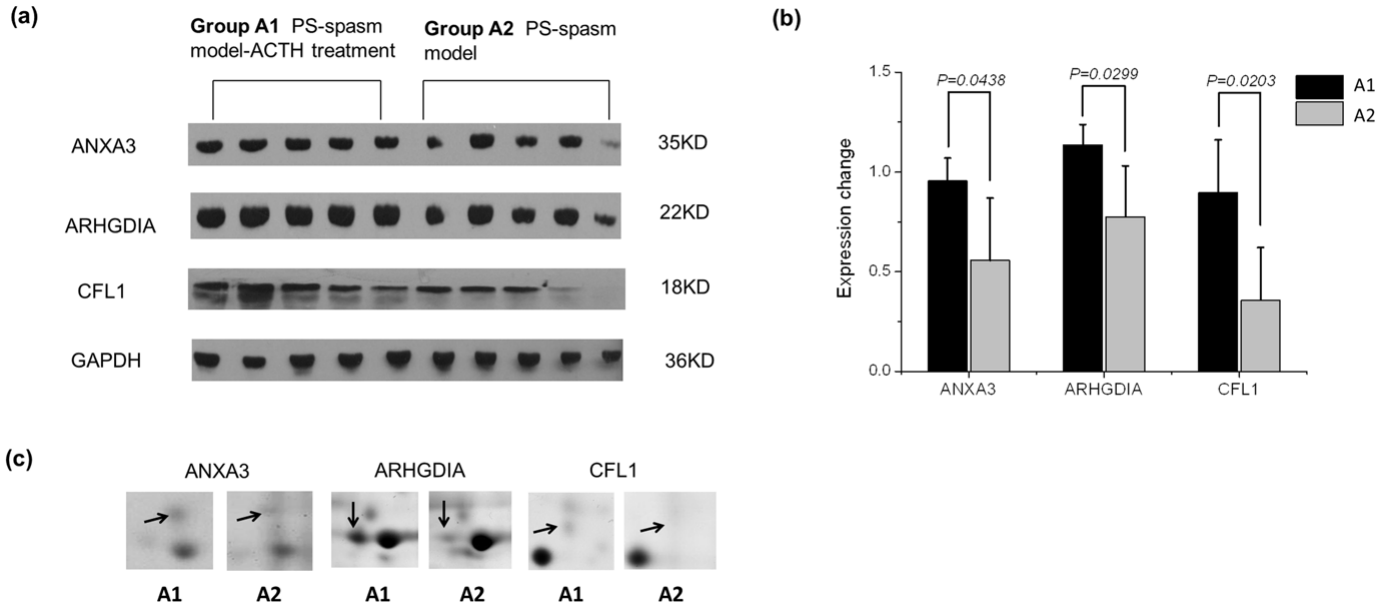
Increased expression of ANXA3, ARHGDIA, and CFL1 in the PS-spasm model-ACTH treatment (Group A1). (a) The proteins were analyzed using 12% (SDS–PAGE) and western blot analysis. GAPDH was used for normalization. (b) The intensities of ANXA3, ARHGDIA, and CFL1 expression from the immunoblots are shown in (a), as quantified using densitometric analysis. (c) The spots of ANXA3, ARHGDIA, and CFL1 are marked by arrows on the 2DE gel.

We performed western blot analysis to verify the differential expression between Group A1 (PS-spasm model-ACTH treatment) and Group A2 (PS-spasm model) (Figure 3a). Western blot analyses indicated that annexin A3 (ANXA3), ARHGDIA, and CFL1 were up-regulated in Group A1 (Figure 3b). The results are consistent with those of the 2DE (Figure 3c).

### Functional Analysis

**Figure 4:**
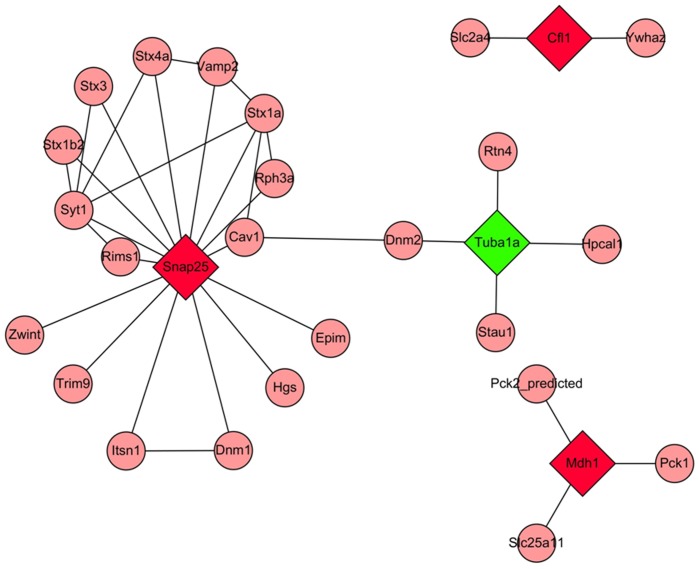
Biological function network map of the differentially expressed proteins. The red diamonds represent up-regulated proteins, green diamonds represent down-regulated proteins, pink circles represent proteins that interact with the differentially expressed proteins, and the lines represent relationships.

According to gene ontology (GO) analysis, the differentially expressed proteins are involved in protein polymerization, microtubule-based movement, and cellular protein complex assembly, with the functional keywords ‘acetylation,’ ‘microtubule,’ and ‘cytoplasm’ (Table 3). The Cytoscape software was used to construct protein networks. The identified proteins formed three functional modules based on protein–protein interactions (Figure 4). The differentially expressed proteins SNAP-25 and Tuba1a were connected by the proteins caveolin-1 (CAV1) and dynamin-2 (DNM2).

**Table 3:** GO analysis and protein functional keyword analysis.

GO Term	PValue	Genes	Fold Enrichment
GO:0051258∼protein polymerization	0.01753637	TUBB5, TUBA1A	97.05714286
GO:0007018∼microtubule-based movement	0.0437855	TUBB5, TUBA1A	38.43847242
GO:0043623∼cellular protein complex assembly	0.04675995	TUBB5, TUBA1A	35.94708995
**key words**	**PValue**	**Genes**	**Fold Enrichment**
acetylation	6.23E-04	CFL1, TUBB5, DDAH1, ANXA3, ARHGDIA, MDH1	5.759354839
microtubule	0.08350872	TUBB5, TUBA1A	20.19683258
cytoplasm	0.09998266	CFL1, SNAP25, ARHGDIA, MDH1	2.947177286

### ARHGDIA Expression in Epileptic Foci of Infantile Spasm Patients

**Figure 5:**
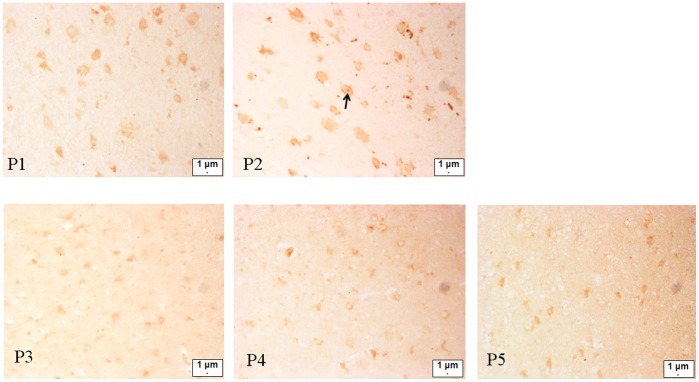
Immunohistochemical staining of ARHGDIA visualized by brown signals in the epileptogenic foci of brain tissue. P1–P5 are the patient IDs. P1 and P2: patients with ACTH treatment, P3–P5: patients without ACTH treatment. From the figure, ARHGDIA expression was more pronounced (no statistical analysis was performed due to the small sample size) in P1 and P2, particularly in P2 who responded to the ACTH treatment. Arrow: possible ARHGDIA expression in the nucleus.

Figure 5 shows ARHGDIA expression in five cases with epileptic foci in the brain. Comparison between patients P1 and P2 who were treated with ACTH and patients P3 to P5 suggests that ARHGDIA expression was more pronounced (no statistical analysis was performed due to the small sample size) in P1 and P2, particularly in P2 who responded to the ACTH treatment. Arrow: Possible ARHGDIA expression in the nucleus.

## Discussion

Based on clinical epidemiology, PS affects offspring health. Animal studies have clearly demonstrated the effects of PS on the brains of offspring [8]. In our previous study, the incidence of infantile spasms was related to prenatal distress [5]. Our research showed that the spasm frequency did not differ between groups B1 and B2, although the spasm latency period in group B1 was longer than that in group B2. This result implies that NMDA-induced spasms are not sensitive to acute pretreatment with glucocorticoids, which is consistent with other reports [4]. We found that maternal stress during pregnancy results in more severe NMDA-induced seizures in the offspring. Furthermore, ACTH treatment reduces and delays the onset of these seizures, which correlates well with the treatment of infantile spasm with ACTH therapy. PS exposure damages the hippocampal neurons and the neuronal ultrastructure of the offspring and alters neurotransmitter receptor expression. Deutsch reported that the dose-related capability of MK-801 to increase the threshold voltage necessary for eliciting tonic hindlimb extension in mice is reduced 24 h after stress (swimming in cold water) [7]. MK-801 is a noncompetitive NMDA receptor antagonist whose action depends on the open configuration of the channel and its interaction with the hydrophobic domain within the ligand-gated ion channel. This finding suggests that stress does not reduce the number of activated or open channels [9].

We analyzed differentially expressed proteins between the PS-spasm model-ACTH treatment and the PS-spasm model using proteomics technology. Eight significantly differentially expressed proteins were identified, with biological functions related to the cytoskeleton, synapses, energy metabolism, vascular regulation, and signal transduction.

### Cytoskeleton

Spot 8, which represents the tubulin α-1A chain, was down-regulated, whereas Spot 2, the isoform 1 of tubulin β-5 chain, and spot 6, CFL1, were up-regulated. Consistent with our results, in a prior study on temporal lobe epilepsy in an animal model and in patients, cytoskeleton proteins significantly changed, which implies that the cytoskeleton is associated with the pathogenesis of epilepsy. The cytoskeletal structure is complex and serves to control cellular shape, membrane protein distribution, control of intracellular trafficking mechanisms, and generation and motility of growth cones, spines, and dendrites [10]. Tubulin α 1A mutations are associated with type I lissencephaly and are subsequently found in children with mental retardation and brain malformations and exhibit a wide spectrum of severities [11]–[12]. Type I lissencephaly is significantly correlated with infantile spasms, with infantile spasms occurring in more than 90% of type I lissencephaly cases [13]–[14]. Greene et al. reported that the tubulin α-1A chain is up-regulated in pilocarpine-induced temporal lobe epilepsy based on proteomics analysis [15]. These findings imply that the change in tubulin α-1A chain is related to ACTH treatment.

Cofilins are actin-binding proteins that disassemble actin filaments. They are widely distributed in various tissues. Three actin-depolymerizing factors (CFL1, m-cofilin, and actin depolymerization factor) are found in mice and humans. CFL1 is distributed in non-muscle tissue, particularly in the brain and the liver, and is associated with neuronal migration disorders and cell cycle control in the cerebral cortex. Many neuronal disorders, such as lissencephaly, epilepsy, and schizophrenia, are caused by the abnormal migration of neurons in the developing brain. The main defects observed in CFL1 mutant embryos include impaired delamination and migration of neural crest cells, which affect the development of neural crest-derived tissues [16]. CFL1 knockout mice exhibit failure of neural tube closure and die *in utero* [17]. In our study, CFL1 up-regulation in the ACTH treatment group suggests that ACTH improves neuronal migration.

### Synaptic Proteins

Spot 1, which corresponds to SNAP-25, was up-regulated. SNAP-25 is a plasma membrane protein that, together with syntaxin and the synaptic vesicle protein VAMP/synaptobrevin, forms the soluble *N*-ethylmaleimide–sensitive factor attachment protein receptor docking complex that regulates exocytosis. SNAP-25 also modulates different voltage-gated calcium channels. Recent genetic studies in humans and some mouse models indicate that alterations in the SNAP-25 gene structure, expression, and/or function directly contribute to these distinct neuropsychiatric and neurological disorders [18]. SNAP-25 stimulates γ-aminobutyric acid (GABA) release during development and it is expressed in the synaptic anterior of mature GABA neurons, involved in neurotransmission, and closely related with epilepsy [19]. SNAP-25 is down-regulated in temporal lobe epilepsy animal models and in patients by proteomic analysis, which implies synaptosome dysfunction [10]. In the present study, SNAP-25 up-regulation suggests the neuroprotective function of ACTH.

### Energy Metabolism

Spot 4, which corresponds to Mdh1, was up-regulated. Mdh1 is a key enzyme in glucose metabolism that catalyzes the reversible conversion of malate into oxaloacetate and plays an important role in various physical cellular activities, such as mitochondrial energy metabolism. Impaired cellular energy metabolism affects brain injury. The role of mitochondrial energy metabolism in excitotoxic perinatal brain injury was assessed by examining the toxicity of aminooxyacetic acid, a mitochondrial malate-aspartate shunt inhibitor. Previous studies suggest that regional impairment of cellular energy metabolism is an important determinant of selective vulnerability to the brain injury induced by NMDA in perinatal rats [20]. Mitochondrial function is a key determinant of neuronal excitability and viability of neurons in a study that examined the chronic generalized seizures induced by picrotoxin in rats. Mdh1 was decreased by 20% to 80% [21]. The function of cytosolic Mdh1 was analyzed using RNA interference. The *in vitro* oocyte maturation rate was significantly decreased by 34%, and the rate of blastocyst development was 48.1%, which was significantly lower than that in the control. The results indicate that Mdh1 is an essential factor for oocyte maturation and embryo development in mice [22]. Therefore, Mdh1 plays an important role in both energy metabolism and embryonic development. In the ACTH-treatment group, Mdh1 up-regulation improves energy metabolism and the mechanism of ACTH function.

### Vascular Regulation

Spot 5, which corresponds to Ddah1, was up-regulated. This enzyme is primarily found in the cytoplasm. It is highly expressed in vascular endothelial cells and it plays an important role in vascular endothelial regulation. Ddah1 overexpression increases vascular nitric oxide levels and protects against hyperhomocysteinemia-induced alterations in cerebral arteriolar structure and vascular muscle function [23]. Breckenridge examined transgenic Ddah1 knockout mice and found that homozygous Ddah1 null embryos are generated at a low frequency and do not progress through embryonic development. Furthermore, Ddah1 is involved in embryonic development, potentially through its specific effects on the nitric oxide pathways [24]. Ddah1 upregulation in the ACTH treatment group affects blood vessel stability and improves neuroprotection.

### Signal Transduction

Spot 3, which corresponds to ANXA3, was up-regulated. ANXA3 belongs to the lipocortin/annexin family, which bind to phospholipids and membranes in a calcium-dependent manner. Weitzdörfer reported that ANXA3 levels increase in the rat hippocampus at varying developmental time points, which implies that ANXA3 is related to brain development [25]. Chen examined the signaling protein levels in the hippocampus of voluntary and treadmill-exercised rats using a proteomics approach and reported that ANXA3 expression significantly differs under these conditions, which suggests that ANXA3 is involved in signal transduction in hippocampal neurons [26]. Therefore, ACTH treatment is related to brain development and signal transduction.

Spot 7, which represents ARHGDIA, was up-regulated. ARHGDIA belongs to the ras gene superfamily and it is distributed throughout all tissues. Its biological activities include anti-apoptosis, cell movement, negative regulation of cell adhesion, and rho protein signal transduction, among others. ARHGDIA is also associated with neural development and the growth of axons and dendrites. ARHGDIA gene knockout mice showed cognitive deficits, including short-term memory defects, lowered aggression, and altered social behavior. Furthermore, ARHGDIA preferentially impairs the forebrain functions required to form temporal associations, which is similar to mental retardation in humans [27]. Curie performed a neuropsychological and imaging study on patients with mutations in the ARHGDIA gene. Voxel-based morphometric analysis revealed significant changes, mainly in the cerebello-thalamo-prefrontal pathway, in the brain MRIs of patients with ARHGDIA mutations compared with those from normal subjects [28]. The results of the animal models and patient studies suggest that ARHGDIA disorders disrupt the neuronal function. ARHGDIA up-regulation in the ACTH-treatment group suggests that it exerts neuroprotective effects. Hence, we performed a preliminary study on ARHGDIA expression in brain samples from a small but valuable sample of five infantile spasm patients and found differential expression. In the epilepsy foci study of human specimens, our data indicates a similar trend, although the number of samples was limited, i.e., the ARHGDIA expression levels in the ACTH treatment group (P1, P2) were higher than those in the non-treatment group (P3 to P5). In particular, ARHGDIA expression was high in P2, who responded well to treatment. ACTH is the only drug treatment for which long-term infantile spasms outcomes are available. Patients treated early with ACTH had favorable long-term prognoses [29]. In this study, differential ARHGDIA expression was related to ACTH treatment, which indicates that ACTH enhances ARHGDIA expression. However, this study has some limitations. First, a very limited number of samples were studied because of the difficulty in obtaining human specimens because very few infantile spasm patients opt for surgical treatment in China. The five cases underwent the operation because of obvious intracranial lesions and intractable seizures, which do not reflect well in the cryptogenic infantile spasms. Furthermore, no normal human control specimens are available. The specimens comprised epileptic foci from differential lobes (P1, occipital lobe; P2, parietal lobe; P3, parietal lobe; P4, temporal lobe; P5, temporal lobe). Hence, the results are preliminary and further studies will be performed when more samples are obtained.

Using GO analysis, we also found that differentially expressed proteins are involved in protein polymerization, microtubule-based movement, and cellular protein complex assembly. As described above, cytoskeletal stability is closely associated with epilepsy. We found that SNAP-25 and TUBA1A are related through CAV1 and DNM2 by establishing a biological regulatory module. SNAP-25 and CAV1 are present in neurons and colocalize in axonal varicosities. A short-term SNAP-25–CAV1 interaction is possibly involved in the early phase of synaptic potentiation [30]. CAV1 is a scaffolding protein for Src kinase and other components of the endocytic machinery. Src-induced DNM2 phosphorylation is necessary for caveolae release [31]. DNM2 is a member of one of the GTP-binding protein subfamilies, and it binds many proteins that bind to actin and other cytoskeletal proteins. DNM2 also stimulates GTPase activity. These results suggest that SNAP-25 interacts with Tuba1a, which involves neurotransmitter transmission, synaptic reconstruction, and so on.

We also found many differentially expressed proteins (75%, 6/8) associated with the biological function of acetylation in the present study, which indicating that the ACTH treatment mechanism involves the protein acetylation pathway. Protein acetylation regulates a wide range of cellular functions in eukaryotes, particularly via transcriptional control in the nucleus, such as transcription, proliferation, apoptosis, and differentiation. Recently, acetylation was reported to play a significant role in the regulation of metabolic enzymes, metabolic pathways, and metabolic enzyme activity [32]. Deutsch reported that inducing stress in mice (forced swimming in cold water) reduces the antiseizure efficacy of MK-801, which is reversed by sodium butyrate, a histone deacetylase inhibitor that typically promotes gene expression. Sodium butyrate also increases the acetylation of H3 and H4 histone proteins in the hippocampus and the cerebral cortex of mice [33]. These data suggest that protein acetylation can be used to develop epigenetic strategies.

In summary, the differentially expressed proteins include those involved in the cytoskeleton, synapses, energy metabolism, vascular regulation, and signal transduction, which confirm the hypothesis that ACTH modulates the expression and release of several neurotransmitters and neuromodulators. The onset of infantile spasms is age-specific and can be caused by many factors. Cortical malformation during development is one of these important factors. The differentially expressed proteins CFL1, ANXA3, and ARHGDIA are all closely related to brain development, which implies that ACTH therapy promotes brain maturation. The differentially expressed proteins are associated with acetylation, which can be used to develop a therapeutic strategy for treating IS.

## Materials and Methods

### Ethical Statement

The experimental procedures and the animal use and care protocols were approved by the Institutional Animal Care and Use Committee of the General Hospital of the Chinese People's Liberation Army (Approval ID no. 2008B079).

**Table 4:** Clinical characteristics of patients.

ID	Sex	Age	MRI	ACTH treatment	Antiepileptic drugs	Operation
P1	F	16 mo	abnormal signal in the left occipital lobe	ACTH 20 U/day for 20 days by vein injection Six months ago with no response	VAP, TOP	resection of the left occipital epileptic foci
P2	M	14 mo	abnormal signal in the left parietal lobe	ACTH 20 U/day for 16 days by vein injection Seven months ago, seizure-free, but relapsed 19 days after ACTH withdrawal	VAP	resection of the left parietal epileptic foci
P3	M	16 mo	a large area of the right hemisphere malacia	–	TOP, LEV	resection of the right hemisphere
P4	M	4 y	cerebromalacia in the left temporal lobe	–	NZP, TOP, LEV	resection of brain malacia and incision of the corpus callosum
P5	F	3 y	pachygyria of the right occipital lobe	–	CZP, LAM,	resection of the right occipital epileptic foci

LAM, Lamotrigine; LEV, Levetiracetam; SVP, Sodium Valproate; TOP, Topiramate; CZP, Clonazepam; NZP, Nitrazepam.

### Animal and Spasm Models

Three-month-old Wistar rats (weight: 275 g to 345 g, 31 females, 10 males, obtained from the Experimental Animal Center, Capital Medical University) were used in the study. The animals were maintained on a 12 h light/dark cycle with food and water available ad libitum. The female Wistar rats were mated with stud males and the time of appearance of a vaginal plug was considered gestational day 1. The pregnant rats were randomly divided into two groups: Group A, prenatal stress (PS); and Group B, non-prenatal stress. In Group A, the pregnant rats were subjected to physical stress consisting of forced immersion in 4°C water in Plexiglas cylinders. The water was approximately 35 cm deep, so the rats could not stand up without touching the bottom of the cylinder with their tails. After 5 min in the water, the rats were removed and dried for 10 min in a heated container before they were returned to their home cages. The stress was administered daily at 17:00 from day 1 of pregnancy until parturition. Their postnatal 13-day-old pups were randomly divided into two subgroups (n = 15): Group A1, the PS-spasm model-ACTH treatment; and Group A2, the PS-spasm model. In Group A1, the pups were treated with natural, animal-derived ACTH (20 IU/kg, Shanghai Pharmaceutical Co., Ltd., China) by intraperitoneal (i.p.) injection, and were administered NMDA (7 mg/kg, i.p.) after 30 min. In Group A2, the pups were treated with saline, and were administered NMDA (7 mg/kg, i.p.) after 30 min. In Group B, the pregnant rats were not exposed to stress. Their postnatal 13-day-old pups were randomly divided into three subgroups (n = 15): Group B1, the spasm model-ACTH treatment; Group B2, the spasm model; and Group B3, the control. The B1 and B2 pups were administered the same treatment as Groups A1 and A2, respectively. In Group B3, the pups were administered saline (i.p.) as the control. The behaviors of each group were observed continuously for 3 h. High degree of flexion (head and trunk flexion, forelimb, hind limb, and hip flexion) was defined as the onset of spasms. We recorded the latency (after i.p. NMDA administration) to the onset of spasms and the total number of spasms to assess the severity of the spasms.

### Sample Preparation

The brains of five randomly selected rats from each group were removed after decapitation, immediately frozen in liquid nitrogen, and ground into powder. Lysis buffer was added (100 mg in 500 µL) before the addition of 500 µL of protease inhibitors. This was followed by dissolution for 1 h, sonication (4 s/time, 200 W, 12 s intervals), addition of Dnase I and Rnase A, incubation at 4°C, and centrifugation at 40,000×*g* for 1 h. Protein sample quality was then determined using the Coommassie Brilliant Blue (CBB) staining method.

### Two-dimensional Gel Electrophoresis

2DE was performed as reported [34]. Briefly, 1 mg of the protein samples were diluted in rehydration solution (7 M urea, 4% CHAPS detergent, 0.5% IPG buffer v/v, 60 mM DTT) to a final volume of 450 µL and were applied to immobilized pH 3–10 nonlinear gradient (IPG) strips (GE Healthcare, formerly Amersham Biosciences, Uppsala, Sweden) for 16 h. Isoelectric focusing (IEF) analysis was performed using an IPGphor IEF System (GE Healthcare) at 17°C with the following voltage program: 200 V (step and hold for 1 h), 500 V (step and hold for 1 h), 1000 V (step and hold for 1 h), and 8000 V (gradient, total 60 kV•h). Prior to the second-dimensional gel separation, the IPG strips were equilibrated twice for 12 min with gentle shaking in 10 mL of equilibration buffer containing 50 mM Tris-Cl (pH 8.8), 6 M urea, 20% v/v glycerol, and 2% SDS. DTT (1%) was then added to the first equilibration step and 4% iodoacetamide was added to the second equilibration step. Second-dimensional separation (Ettan TMDALT SIX, GE Healthcare) was performed using 12% SDS polyacrylamide gels. The gels were run at 15 mA per gel for 30 min, followed by 30 mA per gel until the bromophenol blue reached the bottom of the gel. The gels were scanned using an Image Scanner (GE Healthcare). Gel analysis of the gels to compare protein content between different samples was performed using Image Master 2-D Platinum software (Amersham Biosciences). Statistical analysis was conducted using SPSS software (Version 13.0, SPSS).

### Mass Spectrometry (MS) Analysis

The protein spots of interests were excised from the CBB-stained gel and were destained in 100 µL of 50 mM ammonium bicarbonate containing 30% CAN for 16 h. The solution was discarded to remove the CBB dye. The dye-washing step was repeated several times until the CBB staining was completely removed. The gel pieces were dehydrated in a SpeedVac evaporator. For trypsin digestion, the gel pieces were rehydrated in 8 µL of trypsin solution, and 25 mM NH_4_HCO_3_ was added until the solution was completely absorbed by the gel. The solution was incubated overnight at 37°C. Then, 100 µL of 5% trifluoroacetic acid (TFA) was added to the dissolved gel pieces, incubated at 37°C for 1 h, and sonicated for 5 min. The suspension was collected and incubated at 37°C for 1 h after the addition of 50% acetonitrile and 2.5% TFA, followed with further sonication for 5 min. The suspension was collected and allowed to dry completely. Matrix-assisted laser desorption/ionization time-of-flight mass spectrometry (MALDI-TOF MS/MS) measurement of the spotted peptide solutions was conducted using an ABI 4800 Proteomics Analyzer (Applied Biosystems, Foster City, CA). The spectra were recorded in reflector mode at a molecular mass range of 900 Da to 3500 Da, with a focus mass of 2000 Da. An International Protein Index (IPI) database search of MS and MS/MS measurements was performed using GPS Explorer software (Applied Biosystems). The peak lists were restricted to Rattus taxonomy, an ms/ms fragment of 0.3 Da, and precursor tolerance of 0.2 Da. A total score over 67 was defined as positive.

### Western Blot Analysis

Protein quantifications of ANXA3, ARHGDIA, and CFL1 were validated using western blot analysis. GAPDH was used as the internal control. A total of 50 µg of the total protein samples was fractionated using electrophoresis through 15% polyacrylamide gels and transferred to a polyvinylidene difluoride membrane (GE Healthcare) following the manufacturer’s instructions. The membrane was probed with the primary antibody, mouse-derived anti-GAPDH antibodies (1∶500 in TBST, Beijing Zhongshan Biotechnology, Zhongshan, China), or rabbit-derived anti-ANXA3 (Bioworld Consulting Laboratories, LLC, Mt. Airy, MD), anti-ARHGDIA (Proteintech, Chicago, IL), and anti-CFL1 (Bioworld) antibodies (1∶500 in TBST) for 1.5 h at room temperature. Then, the membrane was incubated with the HRP-conjugated goat anti-mouse or anti-rabbit secondary antibodies (1∶5000 in TBS-T, Beijing Zhongshan Biotechnology), and incubation at room temperature for 1 h. Chemiluminescence substrate luminal reagent (GE Healthcare) and exposure to X-ray film were used to identify the immunolabeled bands. The optical density of the bands was scanned and was quantified using the Image J program.

### Bioinformatics Analysis

GO analysis and protein functional keyword analysis were performed using DAVID tools based on Fisher’s exact test for GO term enrichment [35]. KEGG (Kyoto Encyclopedia of Genes and Genomes) [36] pathway enrichment analysis was performed using DAVID tools. The *P*-values of the differentially expressed proteins were calculated using the hypergeometric statistical test method and were mapped to the pathway. A *P*-value cut-off of 0.05 was considered significant. The protein interaction networks of the differentially expressed proteins were established based on the Cytoscape platform by searching a public protein–protein interaction database [37].

### Patients

This study was approved by the Institutional Review Board. The paraffin-embedded tissues of five patients were obtained from the Epilepsy Surgery Center of Tsinghua University Yuquan Hospital between 2007 and 2009 (Table 4). All patients were drug-resistant and their imaging findings showed lesions. The decision for surgery was based on the convergent evidence of clinical and electroencephalogram (EEG) recordings and magnetic resonance imaging (MRI) findings. The surgical specimens were analyzed using routine histopathologic examination. The clinical information of the infantile spasm patients is presented in Table 1. Patients P1 and P2 received ACTH treatment. P2 was seizure-free and showed good response to ACTH, but relapsed 19 d after drug withdrawal.

### Immunohistochemistry

The surgical specimens were fixed in formalin via the routine procedure, embedded in paraffin, and cut into 8 mm sections. Then, the sections were deparaffinized, immersed in 3% H_2_O_2_ in darkness for 15 min, and rinsed thoroughly in PBS. The sections were placed in a microwave oven for 20 min in sodium citrate, cooled at room temperature, and blocked with 10% normal goat serum for 12 min. After incubation with primary antibodies, the sections were incubated overnight with anti-ARHGDIA (1∶100) at 4°C. Finally, the sections were incubated with rabbit anti-human IgG and treated with avidin–biotin peroxidase complex for color development. A 3,30-diaminobenzidine kit was used as the chromogen, and the sections were dehydrated in alcohol, washed with xylene, and mounted using gum.

## References

[1] Lux AL, Osborne JP (2004) A proposal for case definitions and outcome measures in studies of infantile spasms and West syndrome: consensus statement of the West Delphi group. Epilepsia. 45: 1416–1428. 10.1111/j.0013-9580.2004.02404.x 15509243

[2] Brunson KL, Avishai-Eliner S, Baram TZ (2002) ACTH treatment of infantile spasms: mechanisms of its effects in modulation of neuronal excitability. Int Rev Neurobiol. 49: 185–197. 10.1016/s0074-7742(02)49013-7 PMC3092432 12040892

[3] Frost JD Jr, Hrachovy RA (2005) Pathogenesis of infantile spasms: a model based on developmental desynchronization. J Clin Neurophysiol 22: 25–36. 15689710 10.1097/01.wnp.0000149893.12678.44

[4] Velisek L, Jehle K, Asche S, Veliskova J (2007) Model of infantile spasms induced by *N*-methyl-d-aspartic acid in prenatally impaired brain. Ann Neurol 61: 109–119. 17315208 10.1002/ana.21082

[5] Shang NX, Zou LP, Zhao JB, Zhang F, Li H (2010) Association Between Prenatal Stress and Infantile Spasms: A Case-Control Study in China. Pediatric Neurology. 42: 181–186. 10.1016/j.pediatrneurol.2009.09.003 20159427

[6] Edwards HE, Dortok D, Tam J, Won D, Burnham WM (2002) Prenatal stress alters seizure thresholds and the development of kindled seizures in infant and adult rats. Horm Behav 42: 437–447. 12488110 10.1006/hbeh.2002.1839

[7] Deutsch SI, Rosse RB, Long KD, Gaskins BL, Burket JA, et al (2008) Sodium butyrate, an epigenetic interventional strategy, attenuates a stress-induced alteration of MK-801's pharmacologic action. Eur Neuropsychopharmacol 18: 565–568. 18164185 10.1016/j.euroneuro.2007.11.004

[8] Van den Hove DL, Steinbusch HW, Scheepens A, Van de Berg WD, Kooiman LA, et al (2006) Prenatal stress and neonatal rat brain development. Neuroscience 137: 145–155. 16242847 10.1016/j.neuroscience.2005.08.060

[9] Deutsch SI, Mastropaolo J, Riggs RL, Rosse RB (1997) The antiseizure efficacies of MK-801, phencyclidine, ketamine, and memantine are altered selectively by stress. Pharmacol Biochem Behav 58: 709–712. 9329063 10.1016/s0091-3057(97)90014-9

[10] Liu XY, Yang JL, Chen LJ, Zhang Y, Yang ML, et al (2008) Comparative proteomics and correlated signaling network of rat hippocampus in the pilocarpine model of temporal lobe epilepsy. Proteomics 8: 582–603. 18186018 10.1002/pmic.200700514

[11] Fallet-Bianco C, Loeuillet L, Poirier K, Loget P, Chapon F, et al (2008) Neuropathological phenotype of a distinct form of lissencephaly associated with mutations in TUBA1A. Brain 131: 2304–2320. 18669490 10.1093/brain/awn155

[12] Jaglin XH, Chelly J (2009) Tubulin-related cortical dysgeneses: microtubule dysfunction underlying neuronal migration defects. Trends Genet 25: 555–566. 19864038 10.1016/j.tig.2009.10.003

[13] Guerrini R (2005) Genetic malformations of the cerebral cortex and epilepsy. Epilepsia 46 Suppl 132–37. 15816977 10.1111/j.0013-9580.2005.461010.x

[14] Kato M (2006) A new paradigm for West syndrome based on molecular and cell biology. Epilepsy Res 70 Suppl 1S87–95. 16806828 10.1016/j.eplepsyres.2006.02.008

[15] Greene ND, Bamidele A, Choy M, de Castro SC, Wait R, et al (2007) Proteome changes associated with hippocampal MRI abnormalities in the lithium pilocarpine-induced model of convulsive status epilepticus. Proteomics 7: 1336–1344. 17366478 10.1002/pmic.200601027

[16] Bellenchi GC, Gurniak CB, Perlas E, Middei S, Ammassari-Teule M, et al (2007) N-cofilin is associated with neuronal migration disorders and cell cycle control in the cerebral cortex. Genes Dev 21: 2347–2357. 17875668 10.1101/gad.434307 PMC1973148

[17] Gurniak CB, Perlas E, Witke W (2005) The actin depolymerizing factor n-cofilin is essential for neural tube morphogenesis and neural crest cell migration. Dev Biol 278: 231–241. 15649475 10.1016/j.ydbio.2004.11.010

[18] Corradini I, Verderio C, Sala M, Wilson MC, Matteoli M (2009) SNAP-25 in neuropsychiatric disorders. Ann N Y Acad Sci 1152: 93–99. 19161380 10.1111/j.1749-6632.2008.03995.x PMC2706123

[19] Tafoya LC, Mameli M, Miyashita T, Guzowski JF, Valenzuela CF, et al (2006) Expression and function of SNAP-25 as a universal SNARE component in GABAergic neurons. J Neurosci 26: 7826–7838. 16870728 10.1523/JNEUROSCI.1866-06.2006 PMC6674219

[20] McDonald JW, Schoepp DD (1993) Aminooxyacetic acid produces excitotoxic brain injury in neonatal rats. Brain Res 624: 239–244. 8252396 10.1016/0006-8993(93)90083-y

[21] Acharya MM, Katyare SS (2005) Structural and functional alterations in mitochondrial membrane in picrotoxin-induced epileptic rat brain. Exp Neurol 192: 79–88. 15698621 10.1016/j.expneurol.2004.11.004

[22] Yoon SJ, Koo DB, Park JS, Choi KH, Han YM, et al (2006) Role of cytosolic malate dehydrogenase in oocyte maturation and embryo development. Fertil Steril 86: 1129–1136. 16962111 10.1016/j.fertnstert.2006.02.105

[23] Rodionov RN, Dayoub H, Lynch CM, Wilson KM, Stevens JW, et al (2010) Overexpression of dimethylarginine dimethylaminohydrolase protects against cerebral vascular effects of hyperhomocysteinemia. Circ Res 106: 551–558. 20019334 10.1161/CIRCRESAHA.109.200360 PMC2831416

[24] Breckenridge RA, Kelly P, Nandi M, Vallance PJ, Ohun TJ, et al (2010) A role for Dimethylarginine Dimethylaminohydrolase 1 (DDAH1) in mammalian development. Int J Dev Biol 54: 215–220. 19757398 10.1387/ijdb.072356rb

[25] Weitzdorfer R, Hoger H, Shim KS, Cekici L, Pollak A, et al (2008) Changes of hippocampal signaling protein levels during postnatal brain development in the rat. Hippocampus 18: 807–813. 18493952 10.1002/hipo.20441

[26] Chen WQ, Viidik A, Skalicky M, Hoger H, Lubec G (2007) Hippocampal signaling cascades are modulated in voluntary and treadmill exercise rats. Electrophoresis 28: 4392–4400. 17963288 10.1002/elps.200700336

[27] Chelly J (1999) Breakthroughs in molecular and cellular mechanisms underlying X-linked mental retardation. Hum Mol Genet 8: 1833–1838. 10469834 10.1093/hmg/8.10.1833

[28] Curie A, Sacco S, Bussy G, de Saint Martin A, Boddaert N, et al (2009) Impairment of cerebello-thalamo-frontal pathway in Rab-GDI mutated patients with pure mental deficiency. Eur J Med Genet 52: 6–13. 18992375 10.1016/j.ejmg.2008.09.003

[29] Stafstrom CE, Arnason BG, Baram TZ, Catania A, Cortez MA, et al (2011) Treatment of infantile spasms: emerging insights from clinical and basic science perspectives. J Child Neurol 26: 1411–1421. 21719797 10.1177/0883073811413129

[30] Braun JE, Madison DV (2000) A novel SNAP25-caveolin complex correlates with the onset of persistent synaptic potentiation. J Neurosci 20: 5997–6006. 10934248 10.1523/JNEUROSCI.20-16-05997.2000 PMC6772581

[31] Shajahan AN, Timblin BK, Sandoval R, Tiruppathi C, Malik AB, et al (2004) Role of Src-induced dynamin-2 phosphorylation in caveolae-mediated endocytosis in endothelial cells. J Biol Chem 279: 20392–20400. 15007081 10.1074/jbc.M308710200

[32] Wang Q, Zhang Y, Yang C, Xiong H, Lin Y, et al (2010) Acetylation of metabolic enzymes coordinates carbon source utilization and metabolic flux. Science 327: 1004–1007. 20167787 10.1126/science.1179687 PMC4183141

[33] Schroeder FA, Lin CL, Crusio WE, Akbarian S (2007) Antidepressant-like effects of the histone deacetylase inhibitor, sodium butyrate, in the mouse. Biol Psychiatry 62: 55–64. 16945350 10.1016/j.biopsych.2006.06.036

[34] Wang J, Gu Y, Wang L, Hang X, Gao Y, et al (2007) HUPO BPP pilot study: a proteomics analysis of the mouse brain of different developmental stages. Proteomics 7: 4008–4015. 17922513 10.1002/pmic.200700341

[35] Huang da W, Sherman B, Lempicki RA (2009) Systematic and integrative analysis of large gene lists using DAVID bioinformatics resources. Nat Protoc 4: 44–57. 19131956 10.1038/nprot.2008.211

[36] Kanehisa M, Goto S, Furumichi M, Tanabe M, Hirakawa M (2010) KEGG for representation and analysis of molecular networks involving diseases and drugs. Nucleic Acids Res 38: D355–360. 19880382 10.1093/nar/gkp896 PMC2808910

[37] Cline MS, Smoot M, Cerami E, Kuchinsky A, Landys N, et al (2007) Integration of biological networks and gene expression data using Cytoscape. Nat Protoc 2: 2366–2382. 17947979 10.1038/nprot.2007.324 PMC3685583

